# Meningoencephalitis caused by *Plesiomonas shigelloides* in a Chinese neonate: case report and literature review

**DOI:** 10.1186/s13052-014-0107-1

**Published:** 2015-01-20

**Authors:** Fang-Qin Xia, Pei-Ning Liu, Yong-Hai Zhou

**Affiliations:** Department of Pediatrics, The Second Affiliated Hospital and Yuying Children’s Hospital of Wenzhou Medical University, 109 West Xueyuan Road, Wenzhou, China

**Keywords:** Neonate, *Plesiomonas shigelloides*, Meningitis, Meningoencephalitis

## Abstract

The most usual presentation of *Plesiomonas shigelloides* infection is an acute gastroenteritis, and extraintestinal manifestations are extremely rare. We reported the first neonate with *P. shigelloides* meningoencephalitis in China and reviewed the twelve cases previously reported. Our report adds further awareness on *Plesiomonas shigelloides* meningoencephalitis in neonate and points out the importance of an early diagnosis and the use of sensitive antibiotics treatment to this fatal infection.

## Background

*Plesiomonas shigelloides*(*Aeromonas shigelloides*)*,* a Gram-negative rod belonging to the family Vibrionaceae, was first isolated in 1947 [1]. This bacterium was previously known as C27, and has been classified in a number of genera, including *Pseudomom, erponia, Scatamonas, Vibrio,* and *Aeromom.* It is a facultative anaerobic, gram-negative, and oxidase-positive bacillus that is found in seawater, soil and animals [2]. The most usual presentation of *P. shigelloides* infection is an acute gastroenteritis [3], and extraintestinal manifestations are extremely rare. So far, 12 cases in which neonates developed *P. shigelloides* meningitis/meningoencephalitis have been described (Table 1) [4-15], and seven of whom died. We reported the first neonate with *P. shigelloides* meningoencephalitis in China and reviewed the twelve previously reported newborns with this infection.

**Table 1:** **Cases of neonatal** ***Plesiomonas shigelloides*** **meningitis/meningoencephalitis**

**Case**	**Sex /Age(d)**	**Weight(kg)/Gestation (weeks)**	**Country**	**Treatment**	**Course**	**Year of publication [Reference]**
1	F/4	2.2/35	ZAF	Rifampicin + Ampicillin	Death	1978 [4]
2	F/1.5	3.7/ND	USA	Ampicillin + Gentamicin	Death	1980 [5]
3	M/2	3.5/41	NA	Penicillin + Gentamicin	Survived	1981 [6]
4	M/4	2.8/39	NA	Ampicillin + Gentamicin	Death	1982 [7]
5	M/2	3.3/43	USA	Ampicillin + Kanamycin	Death	1983 [8]
6	M/1.5	4.0/ND	USA	Cefotaxime	Survived	1988 [9]
7	M/2	2.4/34	BEL	Ampicillin + Netilmicin→	Death	1989 [10]
				Ampicillin + Cefotaxime		
8	M/4	ND/37	GER	Mezlocillin + Netilmicin + Cefotaxime→ Gentamicin + Cefotaxime	Death	1992 [11]
9	F/3	3.4/40	JPN	Cefotaxime	Survived	1994 [12]
10	M/1	3.4/36	CAN	Ampicillin + Gentamicin→	Survived	1996 [13]
				Cefotaxime + Gentamicin		
11	ND/10	ND/ND	CUB	Ampicillin + Gentamicin	Death	1999 [14]
12	F/2	2.8/ND	TUR	Cefotaxime + Amikacin→	Survived	2010 [15]
				Meropenem		

ND: not described; →: change to.

## Case presentation

A female infant weighing 3300 g was born after an uneventful 39 weeks’ gestation. The child was doing well, until the age of 5 days, when she appeared jaundice, poor feeding, reduced movements, frothing at lips, and mild upper gastrointestinal bleeding. She was then transferred to the neonatal intensive care unit in our hospital. On physical examination the baby was found to be severe jaundice, mild tachypnea, slight cyanosis around the oral lips, and hypotonia. Blood and cerebrospinal fluid (CSF) sample were obtained for culture. Laboratory findings revealed a hemoglobin concentration of 175 g/l, a white blood cell(WBC) count of 3.5 × 10^9/l (60% neutrophil), and a platelet count of 94 × 10^9/l. Total bilirubin was 354.7 μmol/l(91.6% indirect bilirubin) and C-reactive protein(CRP) was 67 mg/l. Blood gas analysis revealed severe metabolic acidosis (pH 6.94, BE −18.6 mmol/l) and hyponatremia(126 mmol/l). The CSF contained 385000 × 10^6/l WBC(97% neutrophil) and 30 × 10^6/l erythrocytes. The protein concentration was more than 3 g/1 and the glucose concentration was less than 0.6 mmol/L in the CSF. A Gram stain of the CSF showed Gram-negative rods and meropenem(120 mg ivgtt q12hr) was given. The chest X-ray was normal. Serious hyponatremia happened (117 mmol/l). A blood urea nitrogen concentration of 13.51 mmol/l, a serum creatinine concentration of 105 μmol/l, a glutamic-pyruvic transaminase concentration of 24 IU/l, a Troponin I concentration of 0.11 ng/ml and a brain natriuretic peptide of 7930 pg/ml. Activated partial thromboplastin time was 53.1 s and prothrombin time was 19.6 s.

1.4% NaHCO3, 3% natrichloride and phototherapy was given to deal with the metabolic acidosis, hyponatremia and jaundice. During the course of therapy, upper gastrointestinal hemorrhage appeared and the fresh frozen plasma and losec were given. Then, the infant was in circulatory collapse with a blood pressure of 47/25 mmHg and a firm bulging anterior fontanel. 30 ml/kg of isotonic intravenous fluid was input in 1 hr. Dobutamine and Dopamine were given to improve the circulate (15 ug/kg/min, respectively). Soon after, the baby went into a coma and failure of central respiratory impulses indicated the need for mechanical ventilation. The body temperature of the baby rose to 39°C and convulsive attacks occurred which were treated with Phenobarbital and there was myoclonia. Over the next few hours, the hemoglobin concentration decreased to 95 g/l, the WBC count increased to 14.9 × 10^9/l(83% neutrophil), the platelet count decreased to 38 × 10^9/l, and C-reactive protein increased to 194 mg/l.

On the third day after admission, cultures of both blood and the CSF produced a motile, Gram-negative rod, which was subsequently identified as *P. shigelloides* by its biochemical profile in the Microscan Walk Away 96 system (Dade Behring, Germany). The antibiotic susceptibility testing showed the bacteria to be resistant to ampicillin, but susceptible to imipenem, cefuroxime, cefotaxime, ceftazidime, cefepime gentamicin, ofloxacin, cefoperazone/sulbactam, and aztreonam. Sensitivity to meropenem was not evaluated and the super broad-spectrum β gave n-phenylimide enzymes of this bacteria was negative.

**Figure 1:**
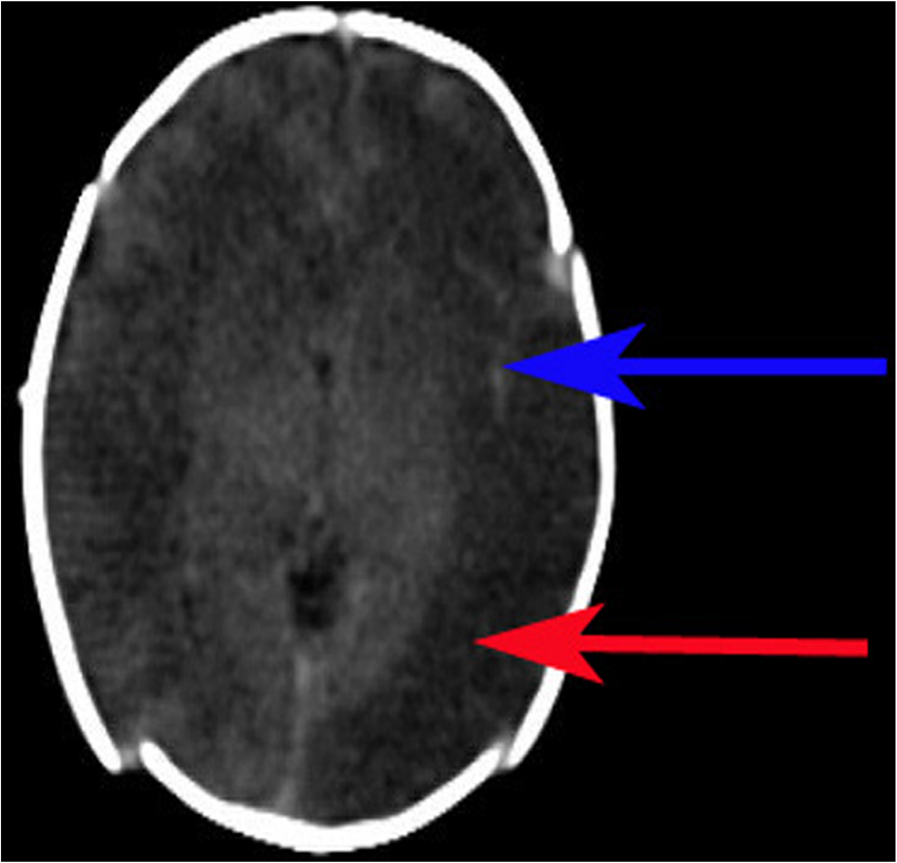
**Computed tomography of head.** Axial CT image show low density lesions in white matter (red *arrow*) and high density in lateral cleft pool (*blue arrow*).

After five days treatment, extensive low density lesions were revealed by cranial computed tomography showing that encephalitis of the whole brain had developed (Figure 1). The CSF was obtained again. The CSF contained 25000 × 10^6/l WBC(97% neutrophil), the protein concentration was 4.12 g/1 and the glucose concentration was 0.18 mmol/l. The second culture of CSF produced *P. shigelloides* again and the result of antibiotic susceptibility testing was consistent with the first one. CRP decreased to 40 mg/l. Stool cultures from this baby was negative for *P. shigelloides*.

On the sixth day after admission, the therapy was stopped after consultation with the parents who worried about the severe sequelae of nerve system, and the neonate died in home few hours later. At the request of the parents, an autopsy was not carried out.

## Discussion and Conclusion

*P. shigelloides* is a very rare causative organism of neonatal meningitis/meningoencephalitis and sepsis. Up to now, the infection has been described in twelve babies, seven (58.3%) of whom died. Our description of a neonate suffering from *P. shigelloides* sepsis and meningoencephalitis is the first case in China. We performed a MEDLINE search for articles published from January 1970 to June 2014. The search terms used were: meningitis or meningoencephalitis and *Plesiomonas shigelloides* or *Aeromonas shigelloides*. Thirteen papers of neonatal with meningitis/meningoencephalitis caused by *Plesiomonas shigelloides*/*Aeromonas shigelloides* are found and two papers described the same case [13,16]. So the relevant features of the 12 reported cases of neonatal with meningitis/meningoencephalitis caused by *P. shigelloides* are summarized in the Table 1.

According to our knowledge, premature infants have an increased susceptibility to infection for the development of whose immune system is immature. However, five of eight babies whom gestational weeks is recorded were term infants. It seems that neither underweight nor premature had a relationship to this disease. It is well known that early onset of neonatal sepsis is rarely seen with meningitis or meningoencephalitis [17]. Most reported cases of neonatal *P. shigelloides* sepsis (12/13, including ours) have been seen in the first five day of life. We consider this point remarkable and open to further discussion. Symptoms began after the first day in all of the cases suggesting a perinatal, rather than transplacental infection. Only three cases have been reported the isolation of the organism from the maternal faeces [6,9,14], and it seems difficult to determine the source and route of this infection notwithstanding a vigorous survey in other cases.

Severe jaundice and/or fever are common first signs in most cases [4,5,9-15], and other common accompanied symptoms including irritability [11,13-15], anorexia [12-15], seizure [5,8,10,13], lethargy [5,15], vasculitic type rash [13], and so on. With the development of disease, circulatory collapse may quickly appear and multiple organs could be affected [8,10]. Hypotonia [4,12], hypermyotonia [11] or endophthalmitis [13] may appear. It is difficult to diagnose the *Plesiomonas shigelloides* infection at early stages by the nonspecific symptoms and signs.

Compared with the other cases, the white blood cells of CSF in our case is the most highest value. Our report of severe metabolic acidosis and hyponatremia in neonatal septicemia and meningoencephalitis due to *Plesiomonas shigelloides* is the first.

Four of five survived neonates were treated with cefotaxime [9,12,13,15]. Five patients treated with ampicillin plus an aminoglycoside or rifampicin died [4,5,7,8,14]. Only one cured patient without neurological deficits was treated with penicillin G and gentamicin first and gentamicin alone subsequently for 15 days [6]. One treated with ampicillin and netilmicin first then changed to ampicillin and cefotaxime died within 2 days of treatment [10]. One treated with mezlocillin and netilmicin first and cefotaxime was added 3 hr after admission then changed to gentamicin and cefotaxime died due to cessation of respiratory therapy at the age of 51 days [11]. The destruction of the CNS happened in two babies [11,13]. By these experiences, we consider it is the key point that third-generation cephalosporins or meropenem is given as early as you can.

Although, in our case immediate, primary therapy was commenced with meropenem-previously used with success in the treatment of bacterial meningitis [14] – we were not able to prevent extensive destruction of the central nervous system. Such result intimated that the invasion of the *Plesiomonas shigelloides* to the brain is very fast.

In conclusion, *Plesiomonas shigelloides* infection in neonate can cause the meningitis/meningoencephalitis and has an impact on cardiovascular system, hematologic system, gastrointestinal system, respiratory system, coagulation system, and so on. The clinical manifestations of sepsis and meningoencephalitis caused by *Plesiomonas shigelloides* in the early stage are similar to those of common infection. However, this disease progresses vary fast and destroys multiple organs. The rate of mortality will be high if the doctor delay to diagnose or take the wrong antibiotics treatment. Although the sensitive antibiotics were given at early, it may be difficult to prevent the extensive destruction of the central nervous system.

## Consent

Written informed consent was obtained from the patient for publication of this Case report and any accompanying images. A copy of the written consent is available for review by the Editor-in-Chief of this journal.

## Abbreviations

CRP: C-reactive protein
CSF: Cerebrospinal fluid
WBC: White blood cell
